# Hope on the horizon: FDA approves eplontersen for hereditary transthyretin-mediated amyloidosis

**DOI:** 10.1097/MS9.0000000000002839

**Published:** 2025-01-09

**Authors:** Insa Binte Anwar, Iqra Furqan Ahmed, Fariha Mahmood, Abdul Haseeb, Khabab Abbasher Hussien Mohamed Ahmed

**Affiliations:** a Liaquat National Medical College, Karachi, Pakistan; b Jinnah Sindh Medical University, Karachi, Pakistan; c Faculty of Medicine, University of Khartoum, Khartoum, Sudan

## Introduction

Hereditary transthyretin-mediated (hATTR) amyloidosis is an autosomal-dominant, disabling, and progressive illness with adult-onset, resulting from Mutations in the transthyretin (TTR) gene which leads to the accumulation of misfolded TTR protein i.e. amyloid which damages the nerve and can cause multisystem disease with a wide range of clinical symptoms leaving the patient with a life span of around 10 years. As per the contemporary data, this hATTR amyloidosis enwraps over 50 000 individuals^[1]^. So far over 120 TTR variations have been linked to hATTR, among which the p. Val30Met mutation is the most prevalent. While most TTR mutations result in a mixed or neuropathic phenotype, other variants usually present with isolated or predominant cardiomyopathy. The p. Val30Met mutation’s clinical appearance is characterized by a length-based neuropathy that mostly affects short fibers. As the disease reaches its peak, it typically appears as a sensory-motor polyneuropathy along with autonomic involvement including altered bowel habits, vomiting and nausea, erectile dysfunction, gastrointestinal abnormalities, postural hypotension, and bladder problems^[2]^.

## Past therapeutic approach and associated adverse effects

Until now, there have been some initial-level advancements in disease therapy. The previously accessible drugs include the TTR tetramer stabilizers (Tafamidis, Vutrisiran, Diflunisal), TTR silencers which include Inotersen, fibril disruptors like doxycycline, Patisiran, monoclonal antibodies, alongside tauroursodeoxycholic acid, alongside invasive therapy like a transplant of liver^[3]^.

Firstly, the NSAID called Diflunisal an anti-inflammatory medication, inhibits the development of TTR amyloid fibrils. Moreover, the therapy of polyneuropathy of hATTR amyloidosis in adults is authorized in the United States of America as well as the European Union where Vutirisiran is injected subcutaneously one single time after three months. When compared to the placebo the drug showed improved results. Secondly, another medication Tafamidis prevents the transthyretin’s tetramers breakdown into monomers by attaching to thyroxine binding domains present on the tetramers. During the medication therapy, there was an improvement in hospitalizations attributable to cardiovascular issues^[4]^. Adding on, the purpose of another drug mentioned Inotersen is to prevent the liver from producing mutant TTR.TTR mRNA generated in the liver cells nucleus is the target of Inotersen, which causes TTR RNA to degrade and obstructs the TTR protein production from mRNA^[3]^.

Previously, another method that was used more frequently was liver transplantation, however, it’s not regarded as the initial choice of therapy due to several reasons including donor accessibility, high cost, and the recipient’s need for continuous immunosuppression^[5]^.

However, the predicted chance of living for the next 10 years in liver transplantation patients for 10 years was 100%^[6]^. Despite the above-mentioned drugs still a lot of development is required. One such example is of Clinical studies being conducted on anti-TTR monoclonal antibodies that suppress the development of fibrils, destroy aggregates, and attack monomers of amyloid. Consequently, patients who take Diflunisal medication for an extended period have a higher likelihood of gastrointestinal and heart-related problems^[3]^. However, one drawback widely reported was pain in limbs and arthritis with Vutirisiran^[7]^. Thirdly, with Inotersen, fever, swelling in the extremities, shivering, muscle ache, altered bowel movements, reduced red blood cell count, clotting disorders, arthralgia, and a lowered platelet count are the symptoms that may be experienced^[3]^. Moreover, patisiran has the harmful effect of causing infusion complications^[8]^.

## Eplontersen

Eplontersen is now licensed in the United States to treat patients with polyneuropathy caused by hATTR amyloidosis (commonly known as ATTRv-PN).

The self-administered auto-injector drug eplontersen is recommended for the medical management of adult polyneuropathy caused by inherited transthyretin-mediated amyloidosis^[9]^. US-based biotechnology business Ionis Pharmaceuticals created the medication. The eplontersen protein reduces the generation of transthyretin (TTR) by utilizing Ionis’ sophisticated LIgand-Conjugated Antisense (LICA) technology. The goal of the method is to deal with ATTR. In December 2023, the US Food and Drug Administration (FDA) approved eplontersen for use in medicine according to the outcomes of Phase III NEURO-TTRansform trial^[10]^.

Eplontersen happens to be an antisense oligonucleotide which inhibits the synthesis of TTR and consequent amyloid deposition by binding to and degrading the wild-type as well as the mutant TTR mRNA. It is designed similarly to the Inotersen, an antisense oligonucleotide that was approved in the past for ATTRv polyneuropathy (ATTRv-PN), and has the same nucleobase sequence^[11]^. A single-dose autoinjector containing 45 mg and 0.8 mL is offered for eplontersen. The suggested dosage for eplontersen normally considered is 45 mg given once a month by subcutaneous injection^[12]^.

Transthyretin-directed antisense oligonucleotide (ASO) called eplontersen is intended to lower TTR protein synthesis. Its transport to hepatocytes is improved by its covalent bond with a ligand that has three N-acetyl galactosamine (GalNAc) residues. ASO compounds become 20–30 times more potent upon coupling with GalNAc, enabling the use of far lower effective dosages^[13]^. Eplontersen works by binding to the TTR mRNA, which causes it to break down and lower blood levels of TTR protein as well as tissue deposits of TTR protein^[9]^.

## Clinical trial; NEURO-TTRansform

NEURO-TTRansform is a multicenter open-label, randomized, phase 3 trial that assesses eplontersen’s safety and effectiveness in patients with ATTRv-PN.2.5. Total of 168 patients having hATTR amyloidosis were enrolled. The research began on 15 January 2020, and completed on 12 July 2023. This study was conducted over 47 locations worldwide including centers in United stated, Argentina, Australia, Brazil, Canada, Cyprus, France, Germany, Greece, Italy, New Zealand, Portugal, Spain, Sweden, Taiwan, and Turkey. In contrast to the external placebo group from the TEGSEDI® (inotersen) NEURO-TTR registrational trial that Ionis completed in 2017, the trial enrolled adult patients with ATTRv-PN Stage 1 or Stage 2.2.5 As per data until week 66, the safety and effectiveness of eplontersen versus an external placebo were compared. the eplontersen treatment group showed improvements in patients with ATTRv polyneuropathy, including considerably low serum transthyretin concentrations, reduced neuropathic impairment, and improved quality of life. serum transthyretin declined by −81.7% compared to −11.2% in patients administered with placebo, as mentioned below in Table 1 ^[13,14]^.

**Table 1:** Clinical trial; NEURO-TTRansform.

Clinical trial details	
Title	A Phase 3 Global, Open-Label, Randomized Study to Evaluate the Efficacy and Safety of ION-682884 in Patients With Hereditary Transthyretin-Mediated Amyloid polyneuropathy
Summary	NEURO-TTRansform is a multicenter, open-label randomized, phase 3 trial that assesses Eplontersen’s safety alongside effectiveness in patients with ATTRv-PN.2.5, which was compared with drug inotersen
Number of patient population	168
Inclusion criteria	Age 18–82 years
	Stage 1 or 2 FAP or Coutinho stage
	Documented TTR gene mutation
	Symptoms consistent with hATTR-PN, including NIS ≥ 10 and ≤ 130
Exclusion criteria	Prior liver transplantation or NYHA classification ≥ 3
	Current or previous treatment with Inotersen, Patisiran, ASOs or Sirna
	Previous treatment must have discontinued ≤2 weeks prior to study day 1
	Tafamidis
	Diflusinal
	Doxycycline
	Abnormal laboratory results:
	UPCR ≥ 1000 mg/g
	Platelets < 125 × 10^9^/L
	eGFR < 45 mL/min/1.73 m^3^
Study duration	January 15, 2020 to July 012, 2023
Study type	Interventional
Phase	phase-3
Disease	Hereditary Transthyretin-Mediated Amyloid Polyneuropathy
Interventional drugs	Eplontersen, inotersen, and the recommended dose of vitamin A
Drug administration	Subcutaneous injection
Results	The eplontersen treatment group showed improvements in patients with ATTRv polyneuropathy, including considerably decreased serum transthyretin concentrations, reduced impaired neuropathy, and improved quality of living.
Limitations	Analysis included a single-group, prospective
	Minor differences in baseline characteristics between the eplontersen and historical placebo groups
	Trial excluded patients with the most severe disease (Coutinho stage 3)

In NEURO-TTRansform, the most frequent adverse effects of eplontersen were decreased vitamin A levels (including vitamin A deficiency, which affected 15% of patients), injection site reactions (7%), proteinuria (8%), vomiting (9%), cataracts (6%), and blurred vision (6%). These reactions were reported in at least 5% of participants. Eplontersen recipients experienced three (2%) major adverse events related to atrioventricular block, including one case of a complete block^[11]^.

Three deaths (1.47%) were documented in the eplontersen group up to week 66; all deaths were seen to be possibly due to ATTRv, while none were thought to be related to the eplontersen medicine^[13]^. Hence, regarding safety, the eplontersen showed comparable or reduced rates of drug discontinuation and treatment-related adverse effects associated with the eplontersen compared to the external placebo. The medication was generally considered to be well-tolerated and safe by the authors, with a few cases of injection site responses (1.4%) and no significant adverse events associated with the medication^[15]^.

## Pharmacokinetics and dynamics

After a single SC dose ranging from 45 to 120 mg, the eplontersen exposure is marginally higher than the dose proportional^[16]^.

Eplontersen peak plasma concentrations occur around 2 hours following a SC dosage. Eplontersen does not interact with medications that are heavily bound to plasma proteins. Moreover, less than 1% of the eplontersen dosage is eliminated as unmodified medication within 24 hours in the urine.

Eplontersen, with around 50 times more potency than Inotersen, suppressed the production of wild-type TTR mRNA in the cell culture of human hepatocytes in a dose-dependent manner. The 3 once-weekly dosage of eplontersen dose-dependently decreased hepatic concentrations of human TTR mRNA in transgenic mice portraying the Ile84Ser variant of the TTR gene in humans; the highest possible dose (6 mg/kg/week) resulted in an ideal reduction of 85% in human TTR mRNA. All dosages of eplontersen were tolerated well, and after one dose equal to 21 mg, it showed good safety profiles. Four subcutaneous doses of 90, 60, or 45 mg given once in every 4 weeks for about 13 weeks resulted in a total decrease in TTR levels of about 90% or more^[17]^.

## Future of hereditary transthyretin-mediated amyloidosis

This innovative drug promises to transform the landscape of treatment for hATTR amyloid polyneuropathy because it is the only medication that is licensed by the FDA where the adult with this particular condition may self-administer using an autoinjector called eplontersen. Furthermore, it was noticed that the therapeutic impact and effectiveness of eplontersen were constant regardless of the illness stage^[15]^. Subsequently, in contrast to the prior placebo, patients with ATTRv polyneuropathy treated with eplontersen showed study results linked with considerably reduced serum transthyretin quantity, decreased neuropathy dysfunction, and a better standard of life. The prevalence of therapy termination owing to the negative effects was low, and 51% of those evaluated had minimal side effects. Both the eplontersen and past placebo trials saw a comparable rate of side effects of thrombocytopenia, the few instances that did arise in the eplontersen group were minor and cleared without ceasing the medication^[13]^. When compared to a typical placebo, eplontersen was linked to steady or better indices of heart anatomy and functioning in individuals having cardiomyopathy^[18]^. Hence, it is anticipated that eplontersen with its minimal side effects and promising results is the new hope for the patients suffering from hATTR amyloidosis.

In summary, this research aimed to explore the effectiveness of a new FDA-approved eplontersen for treating hATTR amyloidosis. Our analysis reveals that previous treatment options had less efficacy and more side effects including limb pain, arthritis, and other infusion-related complications. In contrast, the eplontersen is non-invasive and economical and has fewer adverse effects, implying a promising outcome for its use in hATTR amyloidosis. Conclusively, as per the aforementioned clinical trial, eplontersen is an effective and safe therapy option for patients.

## Data Availability

The data that supports the findings of this study is available with the corresponding author upon reasonable request.
